# T134. EXPOSURE TO NITROGEN DIOXIDE DURING CHILDHOOD IS ASSOCIATED WITH INCREASED RISK OF SCHIZOPHRENIA

**DOI:** 10.1093/schbul/sby016.410

**Published:** 2018-04-01

**Authors:** Sussie Antonsen, Pearl L H Mok, Roger T Webb, Preben Bo Mortensen, John McGrath, Esben Agerbo, Jørgen Brandt, Camilla Geels, Carsten Pedersen

**Affiliations:** 1 Aarhus University, The Lundbeck Foundation Initiative for Integrative Psychiatric Research; 2 The University of Manchester, Manchester Academic Health Science Centre (MAHSC); 3 Aarhus University, The Lundbeck Foundation Initiative for Integrative Psychiatric Research, University of Queensland; 4 Aarhus University

## Abstract

**Background:**

Urban-rural differences in schizophrenia incidence have been reported from numerous published studies. Though as yet unknown, the underlying causes responsible for these differences have been postulated to include urban-rural differences in pollution, diet, infections, stress, or selective migration. Exposure to the air pollutant nitrogen dioxide (NO2) has been linked with increased rates of mortality, lung cancer, and psychotropic medication prescribing. However, no study to date has examined whether NO2 exposure during early childhood is associated with schizophrenia risk.

**Methods:**

Utilizing individual-level information in the rich Danish population-based registers enriched with longitudinal information on residential exposure to air pollution, we investigated the putative link between NO2 exposure during childhood and schizophrenia risk. For each cohort member, exposure to NO2 was estimated longitudinally from birth to 10th birthday, expressed as mean of daily exposures at residence across the first 10 years of life. Incidence rate ratios per 10μg/m3 increase in mean NO2 exposure were estimated using survival analysis techniques. Air pollutant exposures were modelled using the UBM model in 1*1km grids covering Denmark from 1979 onwards.

**Results:**

We observed a dose response relationship between childhood NO2 exposure and elevated risk for developing schizophrenia. Risk increased 1.36 fold (95%CI: 1.31–1.41) per 10μg/m3 increase in mean NO2 exposure during childhood. This association remained materially unaltered when adjusted for potential confounders such as family socioeconomic position and history of severe mental disorders. In absolute risk terms, at age 35 a person exposed to more than 25μg/m3 mean NO2 faced a 1.4% risk of schizophrenia whereas persons exposed to less than 10μg/m3 per day faced a 0.8% risk.

**Discussion:**

This is the first population-based study demonstrating that exposure to NO2 during childhood is linked with elevated schizophrenia risk. The potential mechanism of NO2 on the risk of schizophrenia remains to be identified, however, if causality is proven this finding offers great potential for prevention of some cases of schizophrenia.

